# Supplementary material for: Clinical Implications of Noncoding Indels in the Surfactant-Encoding Genes in Lung Cancer
Source: Cancers (Basel). 2019 Apr 17;11(4):552. doi: 10.3390/cancers11040552 (PMC6520783; doi:10.3390/cancers11040552)
Supplement: Supplementary file 1 [file cancers-11-00552-s001.pdf]

Supplementary Materials: Clinical Implications of Noncoding Indels in the Surfactant-Encoding Genes in Lung Cancer

Takahiro Nakagomi, Yosuke Hirotsu, Taichiro Goto, Daichi Shikata, Yujiro Yokoyama, Rumi Higuchi, Sotaro Otake, Kenji Amemiya, Toshio Oyama, Hitoshi Mochizuki and Masao Omata

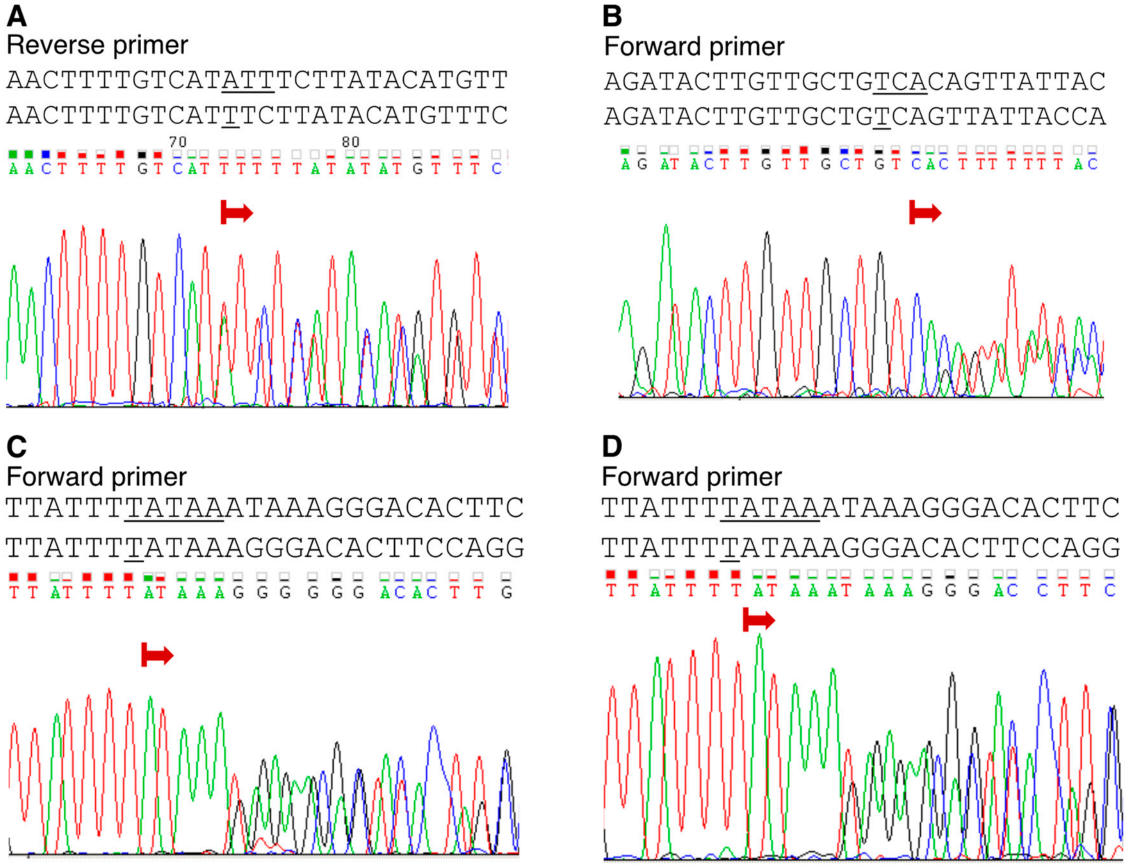

Figure S1. Validation of noncoding indels by Sanger sequencing. **A**, **B**, **C** and **D** represents patient V, XIII, XIV and XVI in Table 2, respectively. Read alignments of targeted sequencing data and Sanger sequencing data were shown in these representative 4 cases. The arrows show the position of the indels.

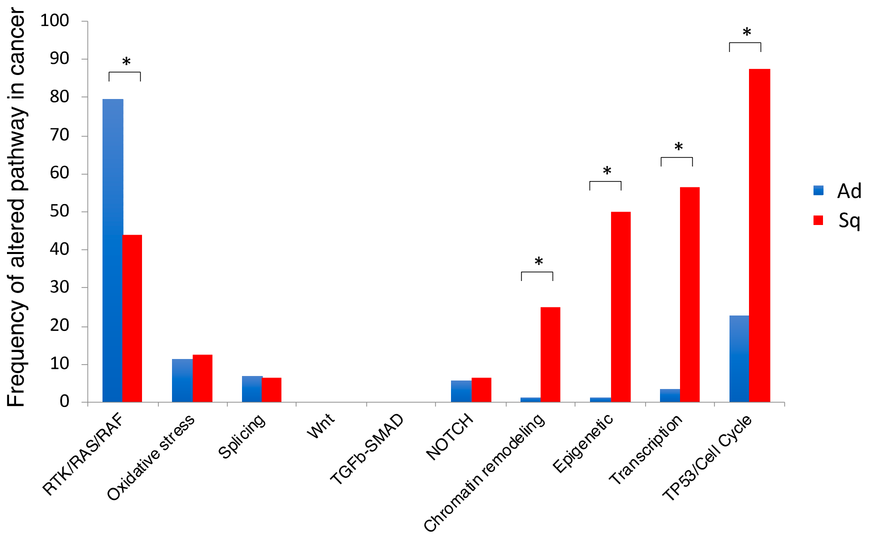

Figure S2. Lung cancer mutation profiles in association with the functional pathways. Difference of mutation profiles between adeno- and squamous cell carcinomas. RAS pathway is predominantly involved in adenocarcinoma, while chromatin remodeling, epigenetic, transcription, and the TP53 pathway are predominantly involved in squamous cell carcinoma. Ad: adenocarcinoma, Sq: squamous cell carcinoma. *, $p < 0.05$.

Table S1. Noncoding indel mutations detected in the samples.

Patient		No. in Table 2	Histology	Locus	Type	Ref	Mutation	Coverage	Gene	AF
1			Ad	chr2:85883742	INDEL	ATTAT	ATTAT/A	1129	*SFTPB*	64%
				chr10:81374890	INDEL	G	G/GTTACTAT	1897	*SFTPA1*	39%
2			Ad	chr8:22021741	INDEL	TTC	TTC/T	458	*SFTPC/BMP1*	51%
3			Ad	chr2:85883771	INDEL	ATC	ATC/A	1400	*SFTPB*	36%
4			Ad	chr8:22022675	INDEL	GAGAA	GAGAA/G	468	*SFTPC/BMP1*	28%
5			Ad	chr10:81375869	INDEL	AAAG	AAAG/A	407	*SFTPA1*	29%
6			Ad	chr2:85884452	INDEL	TTTTTTG	TTTTTTG/T	680	*SFTPB*	11%
7			Ad	chr2:85885000	INDEL	TTC	TTC/T	1203	*SFTPB*	29%
8			Ad	chr2:85885315	INDEL	CAAGA	CAAGA/C	258	*SFTPB*	43%
9			Ad	chr2:85883804	INDEL	GCAT	GCAT/G	1982	*SFTPB*	18%
				chr2:85884441	INDEL	CTCAA	CTCAA/C	954	*SFTPB*	14%
10			Ad	chr10:81374473	INDEL	ATGAT	ATGAT/A	283	*SFTPA1*	20%
11		II	Sq	chr2:85886002	INDEL	CT	CT/C	1124	*SFTPB*	24%
12			Sq	chr2:85884464	INDEL	ATTT	ATTT/A	807	*SFTPB*	37%
13		III	Pleo, AD component	chr2:85883771	INDEL	ATC	ATC/A	901	*SFTPB*	17%
			Pleo, SC component	chr2:85883771	INDEL	ATC	ATC/A	1068	*SFTPB*	20%
**multiple lung cancers**										
14	right	IV	Ad	chr2:85886707	INDEL	GCTT	GCTT/G	926	*SFTPB*	27%
	left	IV	Ad	-	-	-	-	-	-	-
15	right upper	V	Sq	chr2:85885333	INDEL	AAT	AAT/A	353	*SFTPB*	52%
	right lower	V	Sq	-	-	-	-	-	-	-
16	left S1+2	I, VI	Ad	chr2:85884386	INDEL	TTTTTGGGATAA	TTTTTGGGATAA/T	1357	*SFTPB*	25%
				chr2:85885620	INDEL	CT	CT/C	1049	*SFTPB*	24%
				chr2:85888149	INDEL	CAG	CAG/C	1472	*SFTPB*	27%
	left S3	VI	Ad	chr2:85884998	INDEL	CCTT	CCTT/C	1336	*SFTPB*	35%
17	right middle	VII	Ad	-	-	-	-	-	-	-
	right lower	VII	Ad	chr2:85884441	INDEL	CTCAATC	CTCAATC/CTC	1072	*SFTPB*	22%
18	right upper	VIII	Ad	chr2:85883742	INDEL	ATTAT	ATTAT/A	527	*SFTPB*	19%
	right middle	VIII	Ad	-	-	-	-	-	-	-
19	right upper	IX	Sq	-	-	-	-	-	-	-

	left lower	IX	Ad	chr2:85885334	INDEL	ATATGACAAAA GTT ATTTT	ATATGACAAAAGT T ATTTT/A	1281	*SFTPB*	31%
20	right lower	X	Ad	-	-	-	-	-	-	-
	right upper	X	Small	chr2:85884983	INDEL	GAAT	GAAT/G	1134	*SFTPB*	92%
				chr2:85885290	INDEL	TTGTC	TTGTC/T	514	*SFTPB*	91%
21	right	XI	Ad	chr2:85883771	INDEL	ATC	ATC/A	971	*SFTPB*	39%
	left	XI	Ad	chr2:85885294	INDEL	CTGTA	CTGTA/C	320	*SFTPB*	40%
22	left	XII	Ad	chr2:85883742	INDEL	ATTAT	ATTAT/A	735	*SFTPB*	32%
				chr2:85884441	INDEL	CTCAATC	CTCAATC/CTC	599	*SFTPB*	26%
	right	XII	Ad	chr2:85885286	INDEL	CTCTT	CTCTT/C	684	*SFTPB*	13%
23	right upper	XIII	Ad	chr10:81374843	INDEL	TCA	TCA/T	1016	*SFTPA1*	51%
	right lower	XIII	Ad	chr10:81374843	INDEL	TCA	TCA/T	1743	*SFTPA1*	40%
24	right upper	XIV	Ad	chr2:85884451	INDEL	CTT	CTT/C	1330	*SFTPB*	42%
				chr2:85885352	INDEL	TATAA	TATAA/T	1030	*SFTPB*	48%
	left lower	XIV	Ad	chr2:85884451	INDEL	CTT	CTT/C	1244	*SFTPB*	34%
				chr2:85885352	INDEL	TATAA	TATAA/T	1357	*SFTPB*	35%
**mediastinal lung cancers**										
	25	XV	Sq	chr10:81373682	INDEL	T	T/TAGGCCTGACTG	258	*SFTPA1*	28%
				chr2:85885388	INDEL	TAAG	TAAG/T	594	*SFTPB*	18%
	26*	XVI	Ad	chr2:85885352	INDEL	TATAA	TATAA/T	1123	*SFTPB*	41%

Ad, adenocarcinoma; Sq, squamous cell carcinoma; Pleo, pleomorphic carcinoma; SC, sarcomatous; Small, small cell carcinoma; Ref, reference; AF, allele fraction; *, non-surgical case.

Table S2. Clinical characteristics of five cases with cancers of unknown origin.

Case	Case No.in the Paper	Age	Gender	Smoking Habit	Tumor Size (mm)	Diagnostic Procedure	Histology	TTF-1 IHC	Treatmment	Outcomes
1	XV	67	male	smoker	46	surgical biopsy	Sq	negative	Chemo + ICI	28m, alive
2	XVI	61	male	smoker	52	EBUS-TBNA	Ad	negative	Chemo + ICI	22m, alive
3	-	72	male	smoker	41	EBUS-TBNA	Ad	negative	BSC	33m, alive
4	-	67	male	smoker	47	EBUS-TBNA	Sq	negative	Chemo + ICI	32m, alive
5	-	65	female	never	52	EBUS-TBNA	Ad	positive	Chemo	10m, alive

EBUS-TBNA, endobronchial ultrasound-guided transbronchial needle aspiration; TTF-1, thyroid transcription factor 1; IHC, immunohistochemistry; Ad, adenocarcinoma; Sq, squamous cell carcinoma; ICI, immunocheckpoint inhibitor; BSC, best supportive care; m, months.

Table S3. Noncoding regions targeted in the panel.

Chromosome	Start	End	Gene	Chromosome	Start	End	Gene	Chromosome	Start	End	Gene
chr2	85879738	85879832	*SFTPB*	chr2	85887966	85888083	*SFTPB*	chr8	133924552	133924650	*TG*
chr2	85879821	85879906	*SFTPB*	chr2	85888060	85888144	*SFTPB*	chr8	133927249	133927328	*TG*
chr2	85879895	85880002	*SFTPB*	chr2	85888133	85888225	*SFTPB*	chr8	133940203	133940316	*TG*
chr2	85879991	85880100	*SFTPB*	chr2	85888214	85888341	*SFTPB*	chr8	133951593	133951719	*TG*
chr2	85880311	85880440	*SFTPB*	chr2	85888277	85888413	*SFTPB*	chr8	133954960	133955087	*TG*
chr2	85880316	85880443	*SFTPB*	chr2	85888402	85888523	*SFTPB*	chr8	133971172	133971296	*TG*
chr2	85880527	85880600	*SFTPB*	chr2	85888520	85888613	*SFTPB*	chr8	133975767	133975892	*TG*
chr2	85880589	85880695	*SFTPB*	chr4	74270548	74270659	*ALB*	chr8	133978854	133978978	*TG*
chr2	85880684	85880790	*SFTPB*	chr4	74271908	74272027	*ALB*	chr8	133985891	133986014	*TG*
chr2	85881017	85881134	*SFTPB*	chr4	74272641	74272755	*ALB*	chr8	133989506	133989628	*TG*
chr2	85881123	85881246	*SFTPB*	chr4	74274745	74274837	*ALB*	chr8	133990262	133990384	*TG*
chr2	85881235	85881346	*SFTPB*	chr4	74275113	74275227	*ALB*	chr8	133995095	133995216	*TG*
chr2	85881335	85881407	*SFTPB*	chr4	74275345	74275454	*ALB*	chr8	133999379	133999484	*TG*
chr2	85881396	85881481	*SFTPB*	chr4	74276115	74276234	*ALB*	chr8	134009284	134009407	*TG*
chr2	85881513	85881610	*SFTPB*	chr4	74276357	74276435	*ALB*	chr8	134037629	134037765	*TG*
chr2	85881599	85881677	*SFTPB*	chr4	74277261	74277372	*ALB*	chr8	134047463	134047575	*TG*
chr2	85881666	85881787	*SFTPB*	chr4	74279573	74279682	*ALB*	chr8	134053016	134053091	*TG*
chr2	85881808	85881937	*SFTPB*	chr4	74280480	74280563	*ALB*	chr8	134058981	134059055	*TG*
chr2	85881960	85882076	*SFTPB*	chr4	74280873	74280975	*ALB*	chr8	134085450	134085572	*TG*
chr2	85882065	85882143	*SFTPB*	chr4	74281005	74281121	*ALB*	chr8	134095765	134095891	*TG*
chr2	85882132	85882224	*SFTPB*	chr4	74282062	74282182	*ALB*	chr8	134115534	134115654	*TG*
chr2	85882213	85882299	*SFTPB*	chr4	74282680	74282775	*ALB*	chr10	81373540	81373664	*SFTPA1*
chr2	85882672	85882761	*SFTPB*	chr4	74282957	74283034	*ALB*	chr10	81373653	81373780	*SFTPA1*
chr2	85882750	85882868	*SFTPB*	chr4	74283231	74283359	*ALB*	chr10	81373758	81373854	*SFTPA1*
chr2	85882857	85882971	*SFTPB*	chr4	74284154	74284236	*ALB*	chr10	81373843	81373961	*SFTPA1*
chr2	85883066	85883153	*SFTPB*	chr4	74284492	74284585	*ALB*	chr10	81373950	81374041	*SFTPA1*
chr2	85883142	85883259	*SFTPB*	chr4	74284887	74284954	*ALB*	chr10	81374029	81374118	*SFTPA1*
chr2	85883248	85883366	*SFTPB*	chr4	74285283	74285406	*ALB*	chr10	81374150	81374252	*SFTPA1*
chr2	85883342	85883435	*SFTPB*	chr4	74285544	74285663	*ALB*	chr10	81374235	81374340	*SFTPA1*
chr2	85883431	85883536	*SFTPB*	chr4	74286214	74286341	*ALB*	chr10	81374317	81374413	*SFTPA1*
chr2	85883523	85883622	*SFTPB*	chr4	74286988	74287075	*ALB*	chr10	81374447	81374553	*SFTPA1*
chr2	85883613	85883704	*SFTPB*	chr8	22020234	22020363	*SFTPC*	chr10	81374521	81374627	*SFTPA1*
chr2	85883693	85883825	*SFTPB*	chr8	22021335	22021429	*SFTPC*	chr10	81374621	81374717	*SFTPA1*
chr2	85883788	85883896	*SFTPB*	chr8	22021413	22021537	*SFTPC*	chr10	81374706	81374821	*SFTPA1*
chr2	85884323	85884427	*SFTPB*	chr8	22021580	22021707	*SFTPC*	chr10	81374813	81374919	*SFTPA1*
chr2	85884416	85884513	*SFTPB*	chr8	22021699	22021836	*SFTPC*	chr10	81374908	81375030	*SFTPA1*

chr2	85884502	85884621	*SFTPB*	chr8	22021832	22021948	*SFTPC*	chr10	81375019	81375105	*SFTPA1*
chr2	85884603	85884705	*SFTPB*	chr8	22021967	22022084	*SFTPC*	chr10	81375091	81375210	*SFTPA1*
chr2	85884668	85884773	*SFTPB*	chr8	22022078	22022209	*SFTPC*	chr10	81375201	81375318	*SFTPA1*
chr2	85884974	85885073	*SFTPB*	chr8	22022207	22022339	*SFTPC*	chr10	81375307	81375384	*SFTPA1*
chr2	85885277	85885396	*SFTPB*	chr8	22022630	22022764	*SFTPC*	chr10	81375370	81375468	*SFTPA1*
chr2	85885385	85885516	*SFTPB*	chr8	22022635	22022771	*SFTPC*	chr10	81375457	81375569	*SFTPA1*
chr2	85885480	85885605	*SFTPB*	chr8	22022849	22022982	*SFTPC*	chr10	81375563	81375671	*SFTPA1*
chr2	85885598	85885730	*SFTPB*	chr8	22022853	22022987	*SFTPC*	chr10	81375632	81375726	*SFTPA1*
chr2	85885951	85886045	*SFTPB*	chr8	22023154	22023275	*SFTPC*	chr10	81375820	81375929	*SFTPA1*
chr2	85886038	85886121	*SFTPB*	chr8	22023260	22023370	*SFTPC*	chr10	81375912	81376024	*SFTPA1*
chr2	85886110	85886238	*SFTPB*	chr8	22023366	22023456	*SFTPC*	chr10	81376013	81376105	*SFTPA1*
chr2	85886227	85886334	*SFTPB*	chr8	133893066	133893168	*TG*	chr10	81376094	81376163	*SFTPA1*
chr2	85886279	85886401	*SFTPB*	chr8	133898492	133898609	*TG*	chr10	81376238	81376358	*SFTPA1*
chr2	85886397	85886475	*SFTPB*	chr8	133899070	133899195	*TG*	chr10	81376312	81376430	*SFTPA1*
chr2	85886473	85886591	*SFTPB*	chr8	133902832	133902954	*TG*	chr10	81376423	81376540	*SFTPA1*
chr2	85886580	85886704	*SFTPB*	chr8	133904301	133904429	*TG*	chr10	90426094	90426169	*LIPF*
chr2	85886664	85886797	*SFTPB*	chr8	133906880	133906985	*TG*	chr10	90429248	90429360	*LIPF*
chr2	85886795	85886912	*SFTPB*	chr8	133909290	133909375	*TG*	chr10	90430036	90430157	*LIPF*
chr2	85886892	85887023	*SFTPB*	chr8	133909518	133909634	*TG*	chr10	90431460	90431590	*LIPF*
chr2	85887045	85887147	*SFTPB*	chr8	133916079	133916150	*TG*	chr10	90434793	90434858	*LIPF*
chr2	85887136	85887229	*SFTPB*	chr8	133923890	133924019	*TG*	chr10	90436318	90436441	*LIPF*
chr2	85887919	85888008	*SFTPB*								

© 2019 by the authors. Licensee MDPI, Basel, Switzerland. This article is an open access article distributed under the terms and conditions of the Creative Commons Attribution (CC BY) license (http://creativecommons.org/licenses/by/4.0/).
